# Assessment of the Biological Properties of *N*-Nonsubstituted Succinimides and Their Metallocarbonyl Complexes in Normal and Cancer Cells

**DOI:** 10.3390/molecules31010121

**Published:** 2025-12-29

**Authors:** Michał Juszczak, Paulina Tokarz, Aneta Kosińska, Bogna Rudolf, Katarzyna Woźniak

**Affiliations:** 1 University of Lodz, Faculty of Biology and Environmental Protection, Department of Molecular Genetics, Pomorska 141/143, 90-236 Lodz, Poland; michal.juszczak@biol.uni.lodz.pl (M.J.); paulina.tokarz@biol.uni.lodz.pl (P.T.); 2 University of Lodz, Faculty of Chemistry, Department of Organic Chemistry, Tamka 12, 91-403 Lodz, Poland; aneta.kosinska@chemia.uni.lodz.pl (A.K.); bogna.rudolf@chemia.uni.lodz.pl (B.R.)

**Keywords:** metallocarbonyl complexes, succinimides, cytotoxicity, DNA damage, reactive oxygen species, superoxide dismutase, PBM cells, HL-60 cells

## Abstract

Succinimide derivatives display a broad spectrum of biological activities and are being explored for various medical applications, including the treatment of epilepsy, diabetes, and cancer, as well as cardiovascular and liver diseases. Ongoing research continues to yield new derivatives with promising therapeutic potential. This study evaluates the biological properties of 3-methoxysuccinimide (**1**), 3-butynyloxysuccinimide (**2**), and their metallocarbonyl complexes (*η*^5^-cyclopentadienyl)Fe(CO)_2_(*η*^1^-*N*-(3-methoxysuccinimidato)) (**3**) and (*η*^5^-cyclopentadienyl)Fe(CO)_2_(*η*^1^-*N*-(3-butynyloxysuccinimidato)) (**4**) in normal peripheral blood mononuclear cells (PBM) and HL-60 leukemic cells. We examined cytotoxicity, genotoxicity, oxidative, and antioxidative potential of these compounds. Succinimides and their complexes exhibited low cytotoxicity in both cell lines in the concentration range 3–50 μM. At 100 μM, only 3-methoxysuccinimide (**1**) reduced PBM cell viability, while all compounds significantly decreased HL-60 cell viability at this concentration. We also showed that all compounds caused a minor concentration-independent increase in DNA damage level. Interestingly, complex **3** was significantly less genotoxic for HL-60 cells compared to *N*-nonsubstituted analog (**1**). Succinimides **1** and **2** and their metallocarbonyl complexes **3** and **4** demonstrated strong antioxidant properties, especially in HL-60 cancer cells. They also restored SOD activity reduced by oxidative stress in cancer cells.

## 1. Introduction

Succinimides (pyrrolidine-2,5-diones) are a class of compounds characterized by their five-membered cyclic structure, comprising two carbonyl groups and a nitrogen atom. They are mainly known for their anticonvulsant properties and have been thoroughly investigated for their pharmacological effects in this area [1,2,3]. However, succinimides themselves do not show anticonvulsant activity; the substitution of the C3 position of succinimide by one or two alkyl or aryl groups affords potent agents for the management of absence seizures [1]. Succinimides, particularly ethosuximide, are widely employed in the treatment of absence seizures. Their mechanism of action involves inhibiting T-type calcium channels in thalamic neurons, thereby reducing neuronal excitability [4,5].

Beyond their anticonvulsant applications, succinimides are components of active compounds exhibiting diverse biological activities, such as antidiabetic [6,7], antioxidant [8], antifungal [9], and anticancer effects [10,11,12,13]. Many succinimide derivatives act as inhibitors of cholinesterase, α-glucosidase, and α-amylase [14,15]. Succinimide derivatives are also undergoing preclinical testing for their antihypertensive and vasodilator potential [16] and cardioprotective, hepatoprotective, and lipid-lowering effects [17]. Although the findings on the biological activity of succinimides are of great importance, the reports on organometallic succinimide complexes are rather scarce [18].

A previous study by Rudolf et al. demonstrated that the iron, molybdenum, and tungsten half-sandwich succinimide complexes ((*η*^5^-cyclopentadienyl)M(CO)_x_(*η*^1^-*N*-succinimidato) (M = Fe, x = 2; W, Mo, x = 3)) act as reversible inhibitors of the cysteine endoproteinase papain and proved that their binding affinity was highly dependent on the metal center [19]. Wysokiński et al. also examined DNA-damaging effect of the mentioned iron succinimide complex and the maleimide analog ((*η*^5^-cyclopentadienyl)Fe(CO)_2_(*η*^1^-*N*-maleimidato)) in HL-60 cells using the single-cell gel electrophoresis (comet assay). Both investigated complexes were shown to induce *HO-1* gene expression; however, the effect of the succinimide derivative was significantly weaker than that of the maleimide complex [20].

Moreover, Juszczak et al. also investigated the biological activity of ruthenium cyclopentadienyl complexes bearing different succinimide ligands: (*η*^5^-cyclopentadienyl)Ru(CO)_2_(*η*^1^-*N*-methoxysuccinimidato) and (*η*^5^-cyclopentadienyl)Ru(CO)_2_(*η*^1^-*N*-ethoxysuccinimidato) [21,22]. These ruthenium complexes protect cells against H_2_O_2_-induced cell death. The protection was associated with the reduction in the intracellular ROS level and oxidative DNA damage. Additionally, the compounds restored SOD activity under oxidative stress conditions. Our results indicated that ruthenium complexes bearing succinimide ligands have antioxidant activity without cytotoxic effect [21,22].

**Figure 1:**
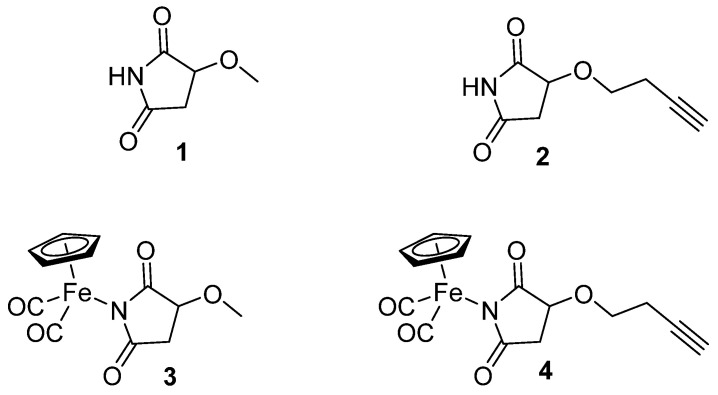
The structures of the ligands (**1**, **2**) and organometallic complexes (**3**, **4**).

In the present study, we focused on iron succinimide derivatives (*η*^5^-cyclopentadienyl)Fe(CO)_2_(*η*^1^-*N*-(3-methoxysuccinimidato)) (**3**) and (*η*^5^-cyclopentadienyl)Fe(CO)_2_ (*η*^1^-*N*-(3-butynyloxysuccinimidato)) (**4**) [23,24], as well as the succinimides that served as their ligands (3-methoxysuccinimide (**1**), 3-butynyloxysuccinimide (**2**)) (Figure 1). This work explores the effect of succinimidato ligands (**1**, **2**) on the biological activity of their metal complexes (**3**, **4**). Imidato ligands often exhibit biological activity that could be comparable or different from that of their corresponding metal complexes [25]. Nevertheless, forming a metal complex can generally offer beneficial effects in terms of selective toxicity towards cancer cells and can lead to a modified mechanism of action.

Therefore, we investigated the effect of compounds **1**–**4** on cell viability, DNA damage, ROS generation and scavenging, and SOD activity. For our study, we selected normal peripheral blood mononuclear cells (PBM) and cancer cells from the acute promyelocytic leukemia (APL) cell line HL-60. HL-60 is notable in research because it includes specific mutations of *c-myc*, *p53*, and *N-ras* oncogenes. It also differentiates into various myelomonocytic cells in vitro. However, these cells often lack some features found in their normal counterparts [26].

## 2. Results and Discussion

### 2.1. Cell Viability

**Figure 2:**
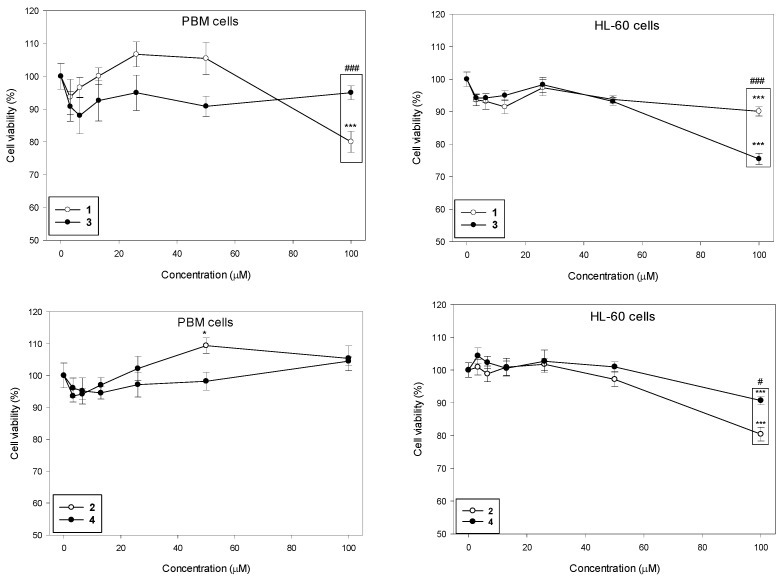
Effect of compounds **1**–**4** on the viability of PBM and HL-60 cells. The viability for individual samples was calculated relative to the negative control (untreated cells) ± SD. Cell viability in the control was taken as 100%, n = 6, * *p* < 0.05, *** *p* < 0.001 vs. negative control; ^#^ *p* < 0.05, and ^###^ *p* < 0.001 vs. complex.

We determined the viability of PBM and HL-60 cells after 24 h incubation with succinimides **1** and **2** and their complexes **3** and **4** in a wide range of concentrations from 3 to 100 µM (Figure 2). Our results indicate that the succinimides and their complexes exhibit no cytotoxicity up to a concentration of 50 µM in both cell types. At the maximum concentration of 100 µM, we observed a statistically significant decrease in PBM cell viability only for succinimide **1** to 80% (*p* < 0.001). In HL-60 cells, all tested compounds caused a statistically significant decrease in cell viability at this concentration; compound **1** and **2** to 90% (*p* < 0.001), compound **3** to 75% (*p* < 0.001), and compound **4** to 80% (*p* < 0.001). Furthermore, we observed a significant difference in the cytotoxicity of succinimide **1** and its complex **3** in normal and cancer cells. In PBM cells, succinimide **1** was significantly more toxic than its complex **3** (*p* < 0.001). In HL-60 cells, we observed the opposite effect: complex **3** was significantly more cytotoxic than ligand **1** (*p* < 0.001). Moreover, we observed that complex **4** was less cytotoxic than succinimide **2** in HL-60 cells (*p* < 0.05) (Figure 2).

It was previously reported that the ruthenium analog of the complex (**3**) (*η*^5^-cyclopentadienyl)Ru(CO)_2_-*N*-methoxysuccinimidato exhibited low toxicity to PBM and HL-60 cells. The IC_50_ dose for both cell types after 24 h of incubation was >250 µM [21]. An increase in cell viability at concentrations up to 250 µM, indicating that this complex stimulates cellular metabolism was observed. Interestingly, after short incubation (2 h) of PBM and HL-60 cells with (*η*^5^-cyclopentadienyl)Ru(CO)_2_-*N*-methoxysuccinimidato, a slight decrease in cell viability at concentrations up to 250 µM [21] was noticed.

### 2.2. DNA Damage

**Figure 3:**
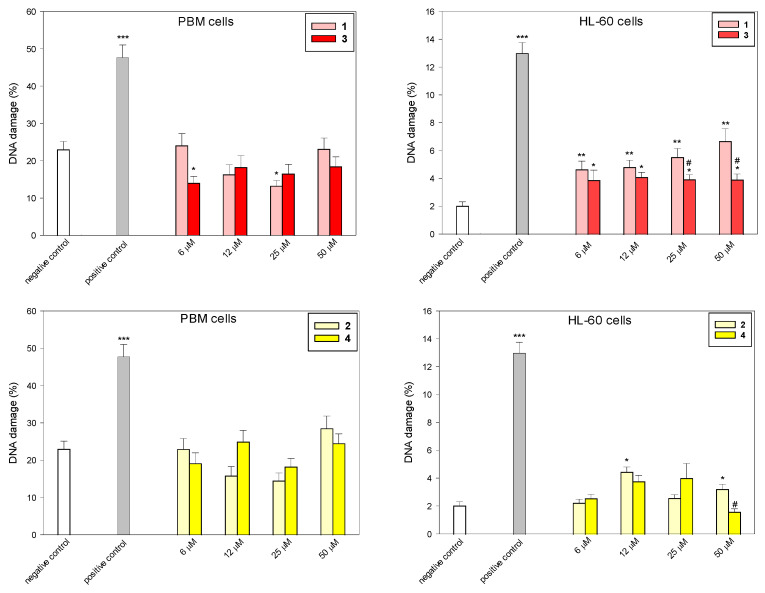
DNA damage induced by compounds **1**–**4** in PBM and HL-60 cells. The figures show mean results ± SEM, n = 100; * *p* < 0.05, ** *p* < 0.01, and *** *p* < 0.001 vs. negative control; ^#^ *p* < 0.05, vs. complex.

**Figure 4:**
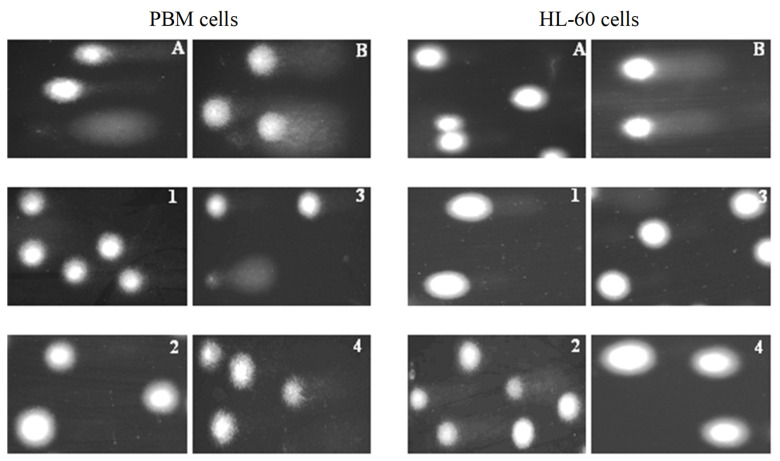
Representative photos of comets obtained in the alkaline version of the comet assay. Effect of medium (negative control) (**A**), H_2_O_2_ (positive control) (**B**) (80 µM, 15 min at 4 °C) (**B**) and **1**–**4** compounds at 25 µM after 2 h incubation of PBM and HL-60 cells at 37 °C.

To determine the level of DNA damage after incubation with succinimides **1** and **2** and their complexes **3** and **4**, we used the comet assay in the alkaline version (Figure 3 and Figure 4). This is a sensitive method that allows for the assessment of the level of DNA strand breaks, including both single- and double-stranded DNA breaks, and alkaline labile sites (ALS). Succinimides, as well as their complexes, induced DNA damage to a small extent in both cell types (Figure 3 and Figure 4). At a concentration of 50 µM, we observed less DNA damage after incubation with complexes compared to damage induced by succinimides. In addition, the effect of complex **3** on DNA in normal and cancer cells is almost unchanged regardless of the dose. On the other hand, DNA damage induced in HL-60 cells by complex **3**, especially at concentrations of 25 µM and 50 µM, is significantly lower than that induced by ligand **1** (*p* < 0.05) (Figure 3). This may indicate a protective role of CO released from the complex against DNA damage [27,28]. However, the mechanism of the CO trigger from metal complexes here is unknown. To avoid the photodegradation of the iron complexes (which leads to CO release) [29], all the biological experiments were carried out in the dark.

In our earlier work, we examined the biological activity of two photoactive iron-based carbon monoxide-releasing molecules (CORMs), i.e., (*η*^5^-cyclopentadienyl)Fe(CO)_2_(*η*^1^-*N*-maleimidato) (complex A) and (*η*^5^-cyclopentadienyl)Fe(CO)_2_(*η*^1^-*N*-succinimidato) (complex B) [20]. It was demonstrated that complex A, but not complex B, was genotoxic to HL-60 cells. DNA damage induced by complex B was efficiently repaired during 2 h repair incubation; in the case of complex A, the process of repair was disturbed. Additionally, complex A induced the expression of the *HO-1* gene, a marker of oxidative stress, to a great extent (over 17-fold for 10 µM), while complex B had a minor effect on *HO-1* gene expression (less than 2-fold induction) [20]. These results indicate a lower cyto- and genotoxic effect of succinimide iron derivatives compared to maleimide ones. The ruthenium analog of the complex **3** (*η*^5^-cyclopentadienyl)Ru(CO)_2_-*N*-methoxysuccinimidato) was not genotoxic to PBM and HL-60 cells during 2 h incubation at a concentration up to 250 µM [21].

### 2.3. ROS Generation and Scavenging

To monitor reactive oxygen species (ROS), we employed the fluorogenic probe 2,7-dichlorodihydrofluorescein diacetate (H_2_DCFDA). The diacetate moieties facilitate its passage through the plasma membrane, where intracellular esterases subsequently hydrolyze them to yield H_2_DCF. This deacetylated form is susceptible to oxidation by a broad range of reactive species, including nitrogen dioxide (^•^NO_2_), carbonate radical anions (CO_3_^•−^), hydroxyl radicals (^•^OH), transition metal ions such as Fe^2+^ and Cu^+^, thiyl radicals like glutathione radical (GS^•^), and enzymatic oxidants such as cytochrome c peroxidase [30].

**Figure 5:**
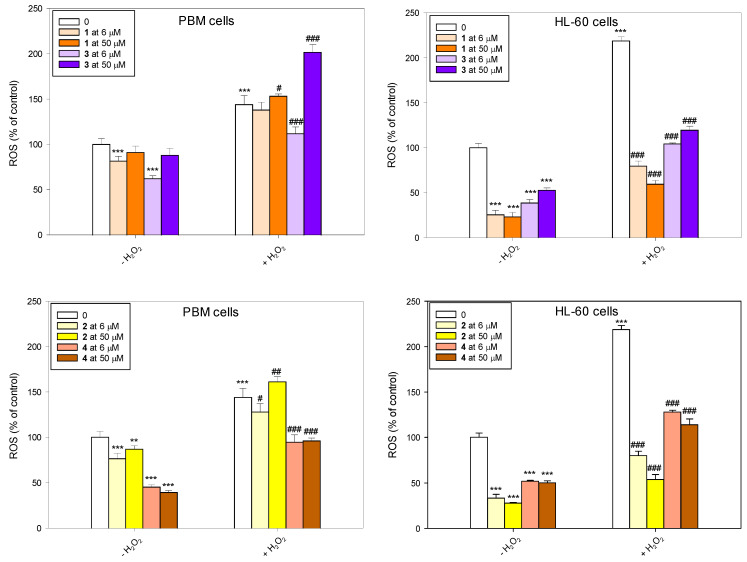
Changes in reactive oxygen species (ROS) level in PBM and HL-60 cells pre-incubated with compounds **1**–**4** for 24 h at 37 °C and then incubated with 1 mM H_2_O_2_ at 37 °C. Each value represents the mean ± SD, n = 6; ** *p* < 0.01, and *** *p* < 0.001 vs. control (−H_2_O_2_); ^#^ *p* < 0.05, ^##^ *p* < 0.01, and ^###^ *p* < 0.001 vs. control (+H_2_O_2_).

Our results indicate that succinimides **1** and **2** and their complexes **3** and **4** do not induce ROS and have strong properties for scavenging free radicals (Figure 5). This effect was particularly pronounced in HL-60 cells, where all tested compounds reduced the level of endogenous ROS. Pre-incubation with succinimides **1** and **2** and their complexes **3** and **4** prevented the increase in ROS level induced by H_2_O_2_. In normal cells, the trend was similar, although the effect was not as pronounced. An exception was complex **3** at a concentration of 50 µM, which caused an increase in ROS induced by H_2_O_2_ and did not reduce the level of endogenous ROS in PBM cells.

### 2.4. SOD Activity

Superoxide dismutase (SOD), which exists in humans in three isoforms—cytosolic Cu/ZnSOD (SOD1), mitochondrial MnSOD (SOD2), and extracellular Cu/ZnSOD (SOD3)—is an oxidoreductase that catalyzes the conversion of superoxide radicals into molecular oxygen and hydrogen peroxide [31]. In addition to SOD, other antioxidant enzymes such as glutathione peroxidase (GPx), glutaredoxins, thioredoxins, and catalase (CAT), in conjunction with non-enzymatic antioxidant compounds, form a fundamental defensive mechanism within the cellular environment.

**Figure 6:**
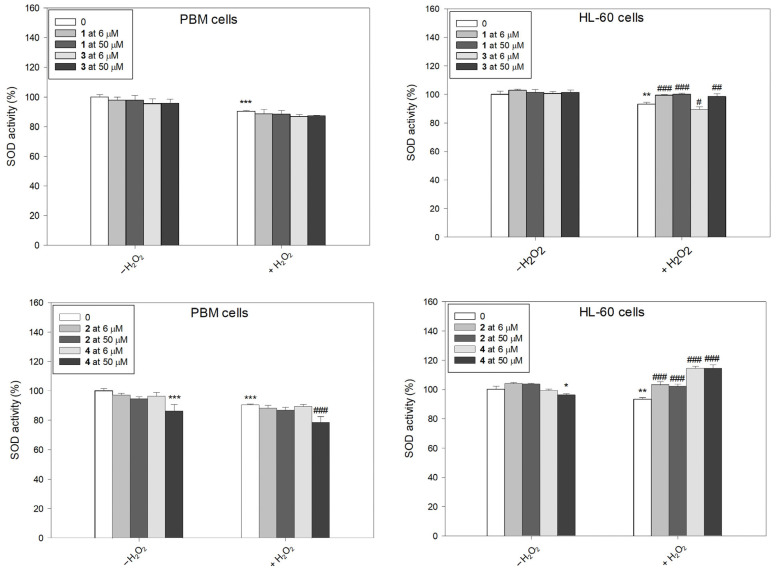
Superoxide dismutase (SOD) activity in PBM and HL-60 cells pre-incubated for 24 h at 37 °C with compounds **1**–**4** and then incubated with H_2_O_2_ at 0.25 mM in the case of PBM cells and 0.1 mM in the case of HL-60 cells. The figure shows the mean results ± SD, n = 3; * *p* < 0.05, ** *p* < 0.01, and *** *p* < 0.001 vs. control (−H_2_O_2_); ^#^ *p* < 0.05, ^##^ *p* < 0.01, and ^###^ *p* < 0.001 vs. control (+H_2_O_2_). Data were normalized to the negative control, which was assigned as 100% of the SOD activity.

After incubation with H_2_O_2_, we observed a decrease in SOD activity in both PBM and HL-60 cells (Figure 6). This result is in agreement with results obtained previously [22,32]. We also observed a decrease in SOD activity after incubation of PBM cells with complex **4** at a concentration of 50 µM. Pre-incubation with succinimide ligands or their complexes, followed by the incubation with H_2_O_2_, led to a clear increase in SOD activity in HL-60 cells. However, complex **3** at 6 µM further decreased in SOD activity (*p* < 0.05). The greatest increase in SOD activity was demonstrated after pre-incubation of HL-60 cells with complex **4** (*p* < 0.001). Interestingly, in normal cells, this complex at 50 µM exerted an opposing effect and caused a further decrease in SOD activity (*p* < 0.001).

It was indicated that the succinimide ligand can be responsible for the antioxidant properties of ruthenium succinimide derivatives [22]. CpRu(CO)_2_**-***N*-(3-methoxysuccinimidato), the ruthenium analog of complex **3** used in this study was investigated [22]. CpRu(CO)_2_-*N*-(3-methoxysuccinimidato) increased the viability of PBM and HL-60 cells decreased by H_2_O_2_, and also altered the HL-60 cell cycle arrested by H_2_O_2_ in the sub-G1 phase. Additionally, it has been shown that the ruthenium complex reduces the levels of ROS and oxidative DNA damage in both normal and cancer cells. It also restored SOD activity reduced by H_2_O_2_ [22]. Complex **3** studied here has similar properties. What is worth emphasizing is the lack of cytotoxic and genotoxic properties up to a concentration of 50 µM iron and ruthenium complexes in normal and cancer cells. At this concentration, the complex **3** increased the level of ROS in normal PBM cells, and the ruthenium analog did not change the level of ROS [26]. In HL-60 cancer cells, both complexes very effectively scavenged ROS induced by 1 mM H_2_O_2_. Moreover, results indicate that the ruthenium complex better reproduces SOD activity after H_2_O_2_ treatment than its iron analog [22].

However, it should be noted that in addition to the succinimide ligand, terminal alkynes may also affect the biological activity of both *N*-nonsubstituted succinimides and the metal complex. Terminal alkynes constitute a specific subgroup within alkynes due to their slightly different properties and reaction behavior.

Many compounds containing terminal alkynes are currently being synthesized and studied for their potential biological properties, including anticancer properties [33,34]. Studies are also being carried out on modifying already known molecules and compounds in this way, including plant-derived compounds. For example, noscapine—a benzylisoquinoline alkaloid that belongs to the phthalideisoquinoline structural subgroup—has been modified for this purpose [35,36]. Although it is selectively toxic to cancer cells, its toxicity is still weak (IC_50_~36 µmol/L against MDA-MB-231 cells, for example) [37]. It was found that a novel analog of noscapine, *N*-propargyl noscapine (NPN), strongly inhibited the viability (IC_50_ = 1.35 ± 0.2 μM) and clonogenicity (IC_50_ = 0.56 ± 0.06 μM) of the TNBC cell line, MDA-MB-231, with robust G2/M arrest. NPN was also found to interact with tubulin, compromising its structural stability and enhancing its affinity for colchicine in vitro. In cellular models, NPN induced an atypical pattern of microtubule disruption, inhibited the reassembly of microtubules after cold-induced depolymerization, and reduced their overall dynamic behavior.

The noscapine analog N-4-CN exhibited the highest cytotoxicity toward the triple-negative breast cancer cell line MDA-MB-231 (IC_50_ = 2.7 ± 0.1 µM) and the lowest effect on normal VERO epithelial cells (IC_50_ = 60.2 ± 3 µM). It disrupted the tertiary structure of tubulin and enhanced colchicine binding. In cellular assays, N-4-CN hyperstabilized microtubules and impaired their recovery following cold-induced depolymerization. These combined effects on tubulin dynamics led to cell cycle arrest and apoptosis, accompanied by increased ROS generation [36].

## 3. Materials and Methods

### 3.1. Chemicals

Succinimides and their iron metallocarbonyl complexes were synthesized as previously described (**3**) [23] (**1**, **2**, **4**) [24]. IMDM and fetal bovine serum (FBS) were obtained from Biowest (Cytogen, Zgierz, Poland). 2′,7′-dichlorofluorescein diacetate (H_2_DCFDA) probe, Hank’s balanced salt solution (HBSS), dimethyl sulfoxide (DMSO), and hydrogen peroxide (H_2_O_2_) were purchased from Sigma-Aldrich (St. Louis, MO, USA). All other chemicals were of the highest commercial grade available. A stock solution of complexes and ligands (10 mM) was dissolved in DMSO and then diluted with medium.

### 3.2. Cell Culture

The HL-60 cell line (human promyelocytic leukemia) was sourced from the American Type Culture Collection (ATCC) and cultured in Iscove’s Modified Dulbecco’s Medium (IMDM) supplemented with 15% fetal bovine serum and antibiotics (100 µg/mL streptomycin and 100 U/mL penicillin). Cells were incubated at 37 °C in a 5% CO_2_ atmosphere and passaged every 2–3 days to maintain logarithmic growth.

Peripheral blood mononuclear (PBM) cells were obtained from buffy coats donated by healthy volunteers at the Central Blood Bank in Lodz, following informed written consent. The buffy coat was diluted 1:1 with PBS and subjected to density gradient centrifugation using Lymphosep (Cytogen, Zgierz, Poland) at 2200 rpm for 20 min with minimal acceleration and deceleration. Isolated cells were washed three times with PBS and resuspended in RPMI 1640 medium [22].

### 3.3. Cell Viability

The cell viability resazurin assay was performed similarly to the method described by O’Brien et al. [38]. Resazurin salt powder was dissolved in sterile PBS buffer. PBM and HL-60 cells were incubated with compounds **1**–**4** at the concentrations of 3.2, 6.5,13, 26, 50, and 100 µM for 24 h at 37 °C in 5% CO_2_. Next, cells were washed with warm PBS. Then, cells were seeded on a 96-well plate at a count of 5 × 10^4^ cells per well for PBM cells and 1.5 × 10^4^ cells per well for HL-60 cells. Next, 10 µL of resazurin salt was added to each well, and the plates were again incubated at 37 °C in 5% CO_2_ for 2 h. After that, fluorescence was measured with the HT microplate reader Synergy HT (Bio-Tek Instruments, Winooski, VT, USA) using λ_ex_ = 530/25 nm and λ_em_ = 590/35 nm. The effect of compounds on cell viability was quantified as the percentage of control fluorescence. PBM cells were incubated for 24 h at 37 °C with 50 µM doxorubicin, and HL-60 cells were incubated for 24 h at 37 °C with 100 nM doxorubicin as positive controls of the method. 

### 3.4. DNA Damage

The comet assay was performed under alkaline conditions to analyze DNA damage in PBM and HL-60 cells, following the procedures described by Singh et al. [39], in modification of Tokarz et al. [40]. Cells were incubated with compounds **1**–**4** at the concentrations of 6, 12, 25, 50, and 100 µM for 2 h at 37 °C. A freshly prepared cell suspension in 0.75% low-melting-point (LMP) agarose (dissolved in PBS) was layered onto microscope slides (Superior, Bad Durkheim, Germany) pre-coated with 0.5% normal-melting-point (NMP) agarose. The embedded cells were lysed at 4 °C for 1 h in a lysis buffer containing 2.5 M NaCl, 0.1 M EDTA, 10 mM Tris, and 1% Triton X-100 (pH 10). After lysis, the slides were placed in an electrophoresis chamber, and DNA was allowed to unwind for 20 min in an alkaline buffer (300 mM NaOH, 1 mM EDTA, pH > 13). Electrophoresis was conducted in a buffer containing 30 mM NaOH and 1 mM EDTA (pH > 13) for 20 min at 0.73 V/cm (28 mA) at 4 °C (with running buffer temperature not exceeding 12 °C). Following electrophoresis, slides were rinsed with water, dried, stained with 2 µg/mL DAPI, and covered with coverslips. To minimize artificial DNA damage, the procedure was performed under subdued light or in darkness. Cells treated with 80 µM H_2_O_2_ for 15 min at 4 °C served as a positive control.

Comet images were captured at 200× magnification using an Eclipse fluorescence microscope (Nikon, Tokyo, Japan) equipped with a ProgRes MF cool monochrome camera (JENOPTIK, Jena, Germany) and analyzed using Lucia-Comet v. 7.3 software (Laboratory Imaging, Prague, Czech Republic). Fifty comets were randomly selected per sample, and DNA damage was quantified as the percentage of DNA present in the comet tail.

### 3.5. Measurement of Reactive Oxygen Species

Intracellular reactive oxygen species (ROS) levels were assessed using the cell-permeable, non-fluorescent probe 2′,7′-dichlorofluorescein diacetate (H_2_DCFDA). Upon entering cells, H_2_DCFDA is deacetylated by esterases and subsequently oxidized to the highly fluorescent 2′,7′-dichlorofluorescein. PBM and HL-60 cells were seeded at densities of 2.5 × 10^6^ and 0.5 × 10^6^ cells/mL, respectively, and treated with compounds **1**–**4** at concentrations of 6 and 50 µM for 24 h at 37 °C in 5% CO_2_. Following treatment, cells were washed twice with HBSS and stained with 20 µM H_2_DCFDA (Sigma-Aldrich, St. Louis, MO, USA) for 30 min at 37 °C in the dark. Cells were then washed again and incubated with 1 mM H_2_O_2_ at 37 °C in the dark. Fluorescence intensity was measured after 30 min at λ_ex_ = 495 nm and λ_em_ = 530 nm using a Synergy HT microplate reader (Bio-Tek Instruments, Winooski, VT, USA). Results were calculated using the formula: (Tx − T_0_/T_0_) × 100, where Tx represents fluorescence at the indicated time and T_0_ the baseline value at the start of measurement [22].

### 3.6. Measurement of SOD Activity

PBM and HL-60 cells were seeded at densities of 3 × 10^6^ cells/mL and 1 × 10^6^ cells/mL, respectively, in 75 cm^2^ cell culture flasks. The cells were incubated with compounds **1**–**4** at the concentrations of 6 and 50 µM for 24 h at 37 °C in 5% CO_2_. HL-60 cells were first washed with PBS and treated with 0.1 mM H_2_O_2_ for 15 min on ice. For PBM cells, 0.25 mM H_2_O_2_ was applied for 15 min at 37 °C [22]. Following treatment, cells were lysed by sonication on ice in 0.5 mL of PBS for 30 s using a 4710 Series Ultrasonic Homogenizer (Cole-Parmer Instrument Co., Chicago, IL, USA). Superoxide dismutase (SOD) activity was determined using the SOD Assay Kit-WST (Dojindo, Kumamoto, Japan) according to the manufacturer’s protocol. Cell lysates were used as the sample solution. For each well, 200 µL of WST working solution was added, followed by 20 µL of enzyme working solution. After thorough mixing, the plate was incubated at 37 °C for 20 min. Absorbance was measured at 450 nm using a Synergy HT microplate reader (Bio-Tek Instruments, Winooski, VT, USA).

### 3.7. Statistical Analysis

Cell viability, ROS levels, and SOD activity are reported as mean values ± standard deviation (SD) based on a minimum of three replicates. Each experiment was performed in two independent replicates. Results from the comet assay are presented as mean ± standard error of the mean (SEM) from two independent experiments. In each independent experiment, 50 comets were counted. Statistical analysis was conducted using the Mann–Whitney test for non-normally distributed data and Student’s *t*-test for normally distributed samples. Differences were considered statistically significant at *p* < 0.05.

## 4. Conclusions

In these studies, we investigated the biological potential of *N*-nonsubstituted succinimides (**1**, **2**) and their metallocarbonyl complexes (**3**, **4**) in normal peripheral blood mononuclear PBM and HL-60 cancer cells. We observed that the compounds were slightly cytotoxic and genotoxic to normal PBM and HL-60 cancer cells. However, at the maximum concentration of 100 µM, all compounds caused a significant decrease in the viability of HL-60 cancer cells. We also showed that all tested compounds caused a very slight, concentration-independent increase in DNA damage. Interestingly, complex **3** induced significantly lower DNA damage in HL-60 cells compared to succinimide **1**. Succinimides **1** and **2** and their metallocarbonyl complexes **3** and **4** do not induce ROS and exhibit strong ROS scavenging properties, especially in HL-60 cancer cells. In cancer cells, all compounds increase the activity of SOD decreased by H_2_O_2_. The results obtained in this study show that succinimides **1** and **2** and their metallocarbonyl complexes **3** and **4** have strong antioxidant properties with low cyto- and genotoxicity in normal and cancer cells. The ruthenium analog of complex **3** (*η*^5^-cyclopentadienyl)Ru(CO)_2_(*η*^1^-*N*-methoxysuccinimidato), studied earlier, shows similar antioxidant properties, suggesting that the succinimide ligand rather than the metal center is responsible for these properties.

## Data Availability

The original contributions presented in this study are included in the article. Further inquiries can be directed to the corresponding author.
